# The EU AI Act: implications and compliance guidance for healthcare facilities

**DOI:** 10.3389/fdgth.2026.1808373

**Published:** 2026-06-10

**Authors:** Fabio Dennstädt, Janna Hastings, Paul Windisch, Aleksa Jovanovic, Tijana Žunić Marić, Sarah Brüningk, Daniel M. Aebersold, Antje Knopf, Nikola Cihoric

**Affiliations:** 1 Department of Radiation Oncology, Inselspital, Bern University Hospital and University of Bern, Bern, Switzerland; 2 School of Medicine, University of St. Gallen, St. Gallen, Switzerland; 3 Institute for Implementation Science in Health Care, University of Zurich, Zurich, Switzerland; 4 Swiss Institute of Bioinformatics, Lausanne, Switzerland; 5 Department of Radiation Oncology, Cantonal Hospital Winterthur, Winterthur, Switzerland; 6 Wemedoo AG, Steinhausen, Switzerland; 7 Zunic Law, Belgrade, Serbia; 8 Faculty of Medicine, Department Digital Medicine, University of Bern, Bern, Switzerland; 9 Sitem Center for Translational Medicine and Biomedical Entrepreneurship, University of Bern, Bern, Switzerland; 10 Department of Radiotherapy and Radiation Oncology, Faculty of Medicine, University Hospital Carl Gustav Carus, Technische Universität Dresden, Dresden, Germany

**Keywords:** AI Implementation, artifiicial intelligence, compliance, digitalization, EU AI Act, guidance, regulation

## Abstract

**Background:**

The European Union AI Act [Regulation (EU) 2024/1689] establishes the first comprehensive legal framework for artificial intelligence. While AI offers transformative potential in healthcare, its deployment introduces risks regarding safety, bias, and accountability. There is currently a lack of practical operational frameworks to help healthcare facilities transition from legal theory to clinical compliance.

**Methods:**

We performed a qualitative regulatory analysis of the EU AI Act, specifically focusing on the obligations of “deployers” (Articles 26, 27, and 50) in clinical settings. The Act's requirements were cross-referenced with established clinical governance standards (e.g., MDR 2017/745 and FUTURE-AI guidelines). A 10-step compliance roadmap was synthesized and exemplified through a hypothetical case study of a high-risk multimodal breast cancer AI system.

**Results:**

The analysis identifies healthcare as a primary focus of the Act, with most clinical AI tools classified as “high-risk”. We established a four-phase implementation framework: (1) Foundational Strategy and Governance, (2) System Analysis & Risk Assessment, (3) Operational Integration, and (4) Ongoing Compliance. Key results include the definition of mandatory Fundamental Rights Impact Assessments (FRIA), requirements for site-specific technical validation, and the necessity of establishing trust through structured human oversight mechanisms to mitigate automation bias.

**Conclusion:**

The EU AI Act necessitates a shift from transactional procurement to a lifecycle-spanning compliance partnership between vendors and hospitals. While the administrative burden is substantial, the Act provides the essential framework for the safe scaling of medical AI. Proactive alignment with these standards, particularly regarding AI literacy and human oversight, is a strategic necessity for healthcare facilities to ensure patient safety and regulatory readiness by the August 2026 enforcement deadline.

## Introduction

Artificial intelligence (AI) is transforming healthcare, from diagnostics and clinical decision support to patient management and resource allocation. The rapid adoption of AI brings new risks related to safety, bias, transparency, and accountability (1–3). The European Union AI Act [Regulation (EU) 2024/1689] (4) establishes a risk-based regulatory framework with direct implications for healthcare providers. Healthcare facilities must now adapt their processes to comply with the Act, or risk substantial penalties and reputational harm.

A recent EU-commissioned study highlights that while AI has the potential to alleviate systemic pressures like aging populations and workforce shortages, its deployment in clinical practice remains slow (5). Legal and regulatory complexities are among the most important barriers for implementation, which underscores the critical need for a compliance roadmap. Despite the transformative potential of AI, the medical community lacks a standardized operational framework to translate the complex legal requirements of the EU AI Act into clinical practice. The general objective of this work is to provide a structured analysis of the EU AI Act's implications specifically for the healthcare sector. Aim is to identify the critical obligations for healthcare facilities and to synthesize these legal mandates into a practical compliance guide. Furthermore, we provide a practical walkthrough of the compliance steps using a hypothetical breast cancer AI system to clarify the specific duties of healthcare administrators and clinicians throughout the technology's lifecycle.

To guide this analysis, the work seeks to answer the following research questions:

- Question 1: “*What are the primary legal and ethical obligations imposed by the EU AI Act on healthcare providers as opposed to AI developers*?”
- Question 2: “*How can existing hospital governance structures be adapted to meet the specific requirements of transparency, human oversight, and risk management mandated by the Act?”*
- Question 3: “*What practical steps must a clinical department take to ensure a high-risk AI system remains compliant throughout its entire operational lifecycle?”*

## Methodology

### Research typology and design

To address these questions, we conducted a normative analysis and a qualitative evidence synthesis of the European Union's legislative framework, cross-referenced with established clinical governance standards. The methodology follows a deductive approach, applying the legal requirements of the EU AI Act to the specific operational environment of healthcare facilities.

### Data sources and legislative basis

The primary legislative basis for this analysis was the EU AI Act [Regulation (EU) 2024/1689] (4). To ensure a comprehensive overview of the “trust architecture” required in medicine, we cross-referenced the Act with:

- **Complementary Regulations:** The Medical Device Regulation (MDR 2017/745) (6) and the General Data Protection Regulation (GDPR 2016/679) (7).
- **Clinical Governance Standards**: The FUTURE-AI international consensus guidelines for trustworthy AI (8).
- **Empirical Evidence**: Official EU Commission studies [e.g., (5)] and peer-reviewed literature regarding AI explainability, bias, and human-computer interaction in clinical settings.

### Analysis procedure and research stages

The methodology comprised four parts, leveraging a multidisciplinary expert panel comprising specialists in clinical oncology, AI research, health informatics, medical law, and AI implementation.

- **Part 1—Literature Review and Legal Analysis:** We conducted a qualitative analysis of the EU AI Act, with a specific focus on isolating the mandates for “deployers” (Articles 26, 27, and 50). This was supported by a targeted review of current scholarly work and EU policy documentation to identify the primary technological, organizational, and legal barriers to AI adoption in healthcare settings.
- **Part 2—Thematic Categorization:** We categorized the specific obligations of the EU AI Act into three primary domains: Governance and Documentation, Human-AI Interaction, and Post-Market Monitoring. These thematic groups were subsequently cross-referenced with existing hospital workflows. This allowed the expert panel to assign regulatory duties to the appropriate departmental structures (IT, Legal, Clinical, and Ethics).
- **Part 3—Framework Synthesis**: Using the literature review, the panel synthesized the legal mandates into a chronological, 10-step compliance roadmap consisting of four operational phases.
- **Part 4—Operational Exemplification (Model Case)**: To exemplify the practical application of the framework, we applied it to a hypothetical case study involving a high-risk multimodal oncology AI system (“BreastCancer-AI”). This allowed for a step-by-step walkthrough of the transition from procurement to clinical oversight and incident reporting.

The guidance was designed to serve healthcare leadership and clinical frontline staff. The scope encompasses the entire lifecycle of high-risk AI systems, focusing on ensuring safe AI deployment and mitigating legal penalties (Art. 99) while ensuring that human-centered clinical judgment remains the primary safeguard for patient safety.

### Research tools

Technical tools used during the research included the Official EU AI Act Compliance Checker (9) and the FUTURE-AI international consensus guideline (8) for benchmarking trustworthy AI principles.

## Results

### Current state of AI integration and trustworthy AI standards

The key insights derived from current research and policy documents are presented in the following, establishing the clinical and ethical context for the AI Act's implementation in healthcare.

A recent study of the EU on AI deployment in healthcare found that despite its potential, AI adoption is hindered by technological, organizational, and legal barriers (5). The EU AI Act creates a harmonized legal framework needed to overcome uncertainty and build trust. By mandating risk management, transparency, and human oversight, it seeks to ensure that this powerful technology ultimately leads to improved patient care.

Previous studies have highlighted that while the technical performance of AI often exceeds human benchmarks, the “black box” nature of deep learning models poses a fundamental challenge to the principle of medical explainability (10). Scholarly work by Amann et al. emphasized that for AI to be ethically viable in healthcare, it must adhere to a framework of “explainability, transparency, and auditability” (11). These principles are fundamental to the EU AI Act. Furthermore, the “FUTURE-AI” consensus provided a foundational international guideline for deployable AI, identifying data fairness and clinical validation as essential pillars (11).

Successfully implementing these measures provides a pathway to mitigate the most important risks associated with AI in healthcare. Within each EU member state, national authorities are responsible for overseeing the implementation and enforcement of the AI Act. While their primary role is regulatory, many are also tasked with providing guidance and support to deployers (like healthcare facilities) to ensure compliance. This might include Guidance Documents, Workshops and Webinars as well as advisory services. In addition to that many private professional agencies and consultancies have emerged (or expanded their services) to help organizations, navigate the complexities of AI Act compliance.

**Figure 1:**
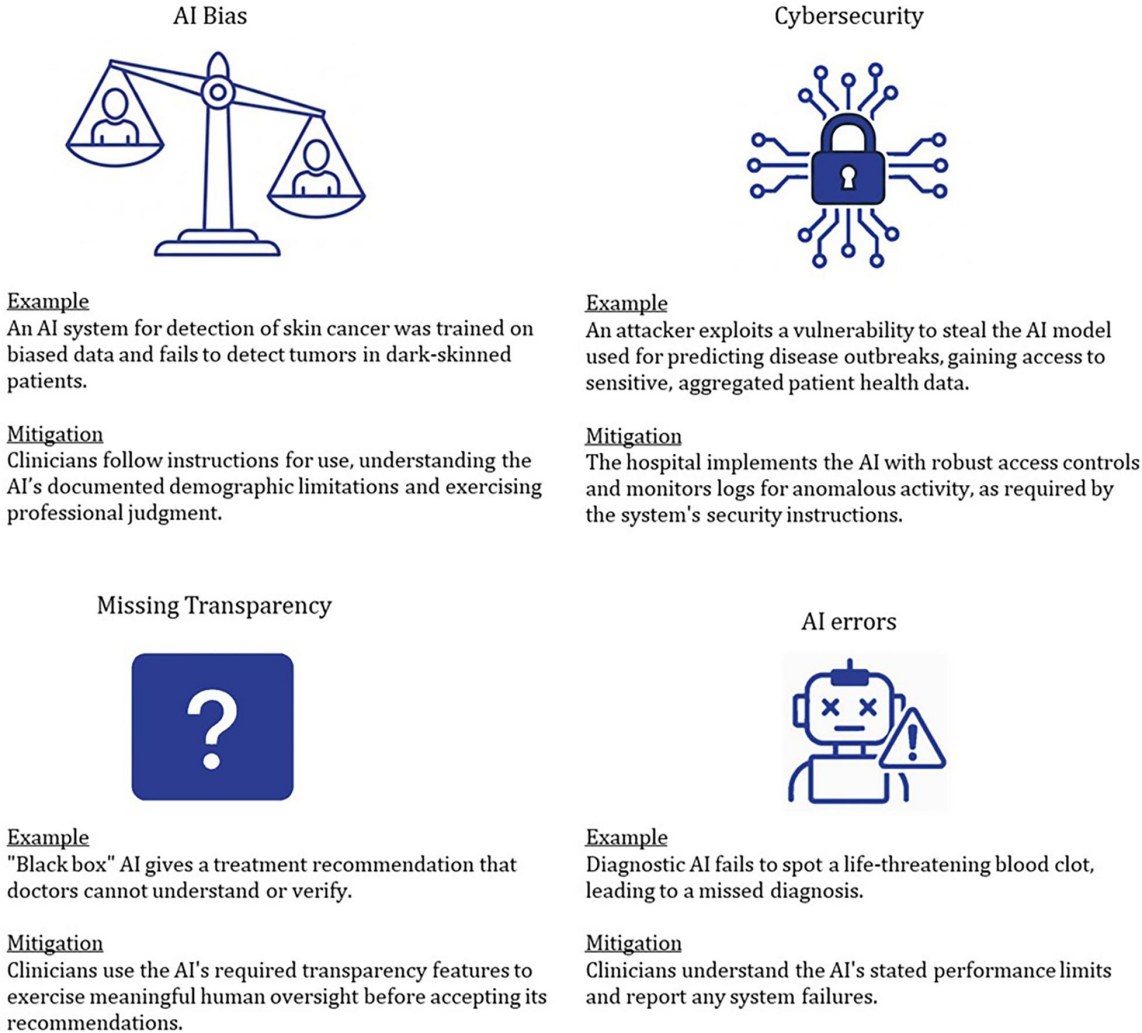
Challenges/issues with particular relevance in AI systems.

AI could fundamentally reshape the landscape of healthcare (12) and AI-driven tools are now integral to several medical disciplines such as radiology or pathology, as well as administrative workflows, increasingly supporting clinical decision-making and operational efficiency (13). More autonomous and generative AI systems including Large Language Models (LLMs) are increasingly being implemented into healthcare with numerous potential applications (14). However, this technological revolution brings with it substantial challenges. Concerns about algorithmic bias, cybersecurity issues, lack of transparency, and erroneous AI systems have become central to the debate on AI in healthcare (1) (see also Figure 1). Each of these domains requires specific mitigation strategies under the EU AI Act. For instance, while algorithmic bias can lead to inequitable access to care, the Act's transparency mandates (Art. 52) serve as a critical check against these errors by requiring clear labeling of AI-generated content.

As AI systems become more complex and autonomous, the risks associated with their deployment are increased (15). For a healthcare facility it is not a viable option to ignore the increasing relevance of AI and the associated regulatory requirements. However, as AI continues to evolve, it is likely that regulatory requirements will become even more complex. For healthcare providers, this means that compliance is not a static goal but an ongoing process, requiring resources, vigilance and adaptability.

### Overview of the EU AI Act

The EU AI Act's (4) primary objective is to ensure that AI systems placed on the EU market are safe, transparent, and respect fundamental rights. The Act applies to all providers, deployers (users), importers, and distributors of AI systems operating within the EU, regardless of whether the AI was developed inside or outside the EU.

The Act defines an “AI system” as “*machine-based system that is designed to operate with varying levels of autonomy and that may exhibit adaptiveness after deployment, and that, for explicit or implicit objectives, infers, from the input it receives, how to generate outputs such as predictions, content, recommendations, or decisions that can influence physical or virtual environments”* [Art. 3 (1)].

Central to the Act is a risk-based regulatory approach. AI systems are classified into four categories: unacceptable, high, limited, and minimal risk (Table 1). “Risk” thereby refers to the potential for an AI system to cause harm to people's health, safety, and fundamental rights.

**Table 1:** Four risk categories defined in the EU AI Act with corresponding regulatory requirements and examples in healthcare.

Risk Category	Regulatory Requirements	Healthcare Example
Unacceptable Risk	Prohibited—These systems are banned due to their threat to safety, fundamental rights, or democratic values. No exceptions are allowed.	An AI system that uses subliminal techniques to manipulate patient behavior, for example, for medication adherence.
High Risk	Strict requirements: Training and AI literacy (Art. 4) Usage in accordance with instructions [Art. 26 (1)] Human Oversight [Art. 26 (2)] Ensure representative Input data [Art. 26 (4)] Monitoring [Arts. 26 (5), 72] Serious incident reporting (Art. 72) Keep Logs [Art. 26 (6)] Registration [for certain deployers; Art. 26 (8), 48] Data protection [Art. 26 (9)] Fundamental Rights Impact Assessment (Art. 27)	AI-powered diagnostic imaging tool, clinical decision support system (CDSS), patient triage algorithm
Limited Risk	Transparency obligations: Users must be informed they are interacting with an AI system [Art. 50 (1)] Clear labeling of synthetic content (Art. 52) Basic instructions for use	AI chatbot providing general health information, symptom checkers for patients on a hospital website.
Minimal Risk	No specific legal requirements voluntary codes of conduct and best practices encouraged.	AI-based appointment scheduling tool, automated inventory management system for hospital supplies.

Unacceptable-risk AI systems are prohibited and include systems used for social scoring, manipulation, human exploitation or social control. In a clinical environment, for example, systems would be considered unacceptable if they use hidden persuasive cues to affect medication adherence or therapeutic choices. However, transparent and clearly communicated gamification algorithms to encourage healthy behavior remain permitted.

High-risk AI systems, which include applications in critical sectors like healthcare, transport, and law enforcement, are subject to strict obligations covering risk management, data governance, technical documentation, human oversight, transparency, and conformity assessment.

Limited- and minimal-risk systems face lighter requirements, primarily focused on transparency and voluntary codes of conduct. Examples of such lower risk systems in a clinical environment might be an AI application for document indexing and connection with other sources, translation of documentation, or assistance with *post-hoc* evaluation of manually curated datasets, such as those used in retrospective clinical studies.

Table 1 provides an overview of the four risk categories defined in the Act with regulatory requirements and examples in healthcare.

Apart from defining risk classes, the Act specifies corresponding obligations, establishes enforcement mechanisms, including market surveillance, penalties for non-compliance, and provisions for regulatory sandboxes to support innovation. By setting standards for the development and use of AI, it aims to balance technological progress with the protection of individual rights and societal values (4, 16).

The high stakes involved in patient safety and public health make healthcare a primary focus for the Act's implementation. A recent policy analysis by the European Parliamentary Research Service emphasizes that while AI can revolutionize clinical workflows, it necessitates a rigid policy framework to manage systemic risks to patient autonomy and data integrity (17). Furthermore, as the Organisation for Economic Co-operation and Development (OECD) has noted, the integration of AI will fundamentally redefine the roles of the health workforce, requiring new international standards for professional accountability and digital competency (18). This aligns with the findings of a comprehensive European Commission study, which identifies regulatory uncertainty as a primary bottleneck for AI adoption in EU hospitals (19). By establishing a harmonized legal framework, the Act aims to mitigate these concerns, providing the “trust architecture” necessary for the safe scaling of medical AI.

### Associated regulatory frameworks

It should be noted that AI systems, if applied in a clinical environment, will in many cases qualify as software as a medical device (SaMD). However, the specific classification depends on the manufacturer's intended purpose and the criteria set out in Annex VIII of the MDR. As such, industrial AI providers bear primary responsibility for conformity assessment, CE marking, and ensuring their systems meet essential requirements before market placement according to Medical Device Regulation (MDR) 2017/745 regulation (20). Medical device AI must satisfy both EU AI Act requirements and MDR 2017/745 standards. CE marking under MDR addresses clinical safety and device performance, while AI Act compliance covers algorithmic transparency, bias mitigation, and fundamental rights protection. These are therefore two complementary but distinct regulatory frameworks.

### Obligations regarding high-risk AI systems

Many AI systems used in healthcare, such as diagnostic tools, clinical decision support systems (CDSS), and patient triage algorithms, are likely to be classified as high-risk (Art. 6, Annex III).

High-risk AI systems are subject to stringent requirements across their lifecycle. Providers (developers) and deployers (users, such as hospitals) have distinct but overlapping obligations. While providers are primarily responsible for the initial conformity assessment, technical documentation, and quality management, deployers hold critical duties for safe implementation. Most of these are provided in Art. 26: Obligations of Deployers of High-Risk AI Systems. These deployer obligations include using AI systems according to instructions, assigning human oversight, monitoring operation, keeping logs, conducting a Fundamental Rights Impact Assessment (FRIA), and cooperating with authorities (Arts. 26–27, 86). An overview of the obligations of providers and deployers is provided in Supplementary Table S1. Good communication and collaboration between provider and deployer are invaluable to ensure implementation is safe, ethical, and in compliance with all relevant regulations.

Transparency is a core principle that imposes duties on both providers and deployers. Deployers, such as hospitals, are responsible for ensuring that patients and staff are informed when they are interacting with an AI system [Art. 50 (1)]. They must also provide affected individuals with a timely, clear, and meaningful explanation of the AI's role in the decision-making process upon request. This explanation should detail the logic and main drivers behind a specific output (Art. 26). This is only possible because providers are obligated to design their systems with the necessary transparency features, such as the capability to generate these explanations and to clearly mark synthetic content, like AI-generated reports, as being machine-made (Art. 52).

### Obligations regarding high-risk AI systems

The EU AI Act is backed by a robust enforcement framework to ensure its obligations are met. National supervisory authorities will be responsible for auditing and enforcing compliance. Penalties for non-compliance can be severe and are levied against the responsible actor (be it the provider, deployer, or another entity in the value chain) depending on the specific violation.

The penalties are tiered based on the severity of the infringement (4, 16):

- For deploying a prohibited AI system, fines can reach up to €35 million or 7% of the company's total worldwide annual turnover for the preceding financial year, whichever is higher [Art. 99 (3)].
- For non-compliance with other key obligations, such as those for high-risk systems, fines can be up to €15 million or 3% of worldwide turnover [Art. 99 (4)].

This enforcement mechanism underscores the legal and financial imperative for healthcare organizations to rigorously adhere to the Act's provisions.

### Implementation timeline and the need for proactive preparation

The EU AI Act follows a phased implementation timeline, which dictates when its various obligations become legally enforceable:

- The ban on unacceptable-risk systems applies from early 2025.
- The comprehensive obligations for high-risk systems become fully enforceable in August 2026.

Waiting to prepare is a substantial strategic risk. A recent study of the EU on AI Deployment in Healthcare revealed that many healthcare facilities feel unprepared for the new regulation (5). The study found that only 26% of hospital representatives surveyed feel ready for the obligations introduced by the Act, citing concerns about the financial and logistical burden of compliance. Certain obligations, while only formally enforceable later, require immediate and sustained effort.

A prime example is the obligation for deployers to ensure “human oversight to natural persons who have the necessary competence, training and authority, as well as the necessary support” [Art. 26 (2)]. This legal duty creates a practical requirement for a sufficiently AI-literate workforce. Clinicians and relevant staff cannot effectively oversee, interpret, or override an AI system without understanding its capabilities, limitations, and potential for erroneous outputs.

Therefore, healthcare facilities have a de facto obligation to begin establishing AI literacy and training programs without delay. This should be viewed as a foundational step to meet future legal requirements, rather than an activity to be postponed until formal enforcement actions and penalties begin.

### Practical implications for hospitals and clinics

For hospital administrators and clinicians, the EU AI Act necessitates fundamental changes in the management and oversight of AI technologies. Complying with the Act requires a structured approach that translates into several key operational imperatives as presented in Table 2.

**Table 2:** Operational imperatives for healthcare facilities under the EU AI Act, distinguishing between mandatory legal obligations and clinical best practices.

Operational Imperative	Description	Status	Relevant Article(s)
AI Inventory & Classification	Systematically identifying and documenting all AI systems to determine risk levels (Unacceptable, High, Limited, Minimal).	Mandatory	Art. 6, Annex III
AI Literacy & Staff Training	Ensuring personnel overseeing AI systems have the necessary technical competence and training to interpret outputs.	Mandatory	Art. 4, Art. 26 (2)
Fundamental Rights Impact Assessment (FRIA)	Assessing the impact of high-risk AI on privacy, non-discrimination, and equitable access to care in the local context.	Mandatory	Art. 27
Human Oversight Mechanisms	Establishing protocols for qualified staff to monitor, intervene, or override AI-driven clinical suggestions.	Mandatory	Art. 14, Art. 26 (2)
Transparency & Communication	Informing patients and staff when AI is used and providing clear explanations of the AI's role in decision-making.	Mandatory	Art. 50, Art. 52
Ongoing Monitoring & Reporting	Implementing continuous performance tracking and reporting serious incidents or malfunctions to authorities.	Mandatory	Art. 26 (5), Art. 72
Formal AI Governance Committee	Establishing a multidisciplinary board (Clinical, IT, Legal, Ethics) to oversee the institutional AI strategy.	Best Practice	—
Vendor Due Diligence & Audits	Verifying provider CE marking/registration and conducting technical audits of model performance on local demographics.	Best Practice	(Supported by Art. 26)
Local Pre-Go-Live Validation	Performing site-specific testing on local hardware and historical data to ensure “real-world” clinical safety and interoperability.	Best Practice	(Supported by Art. 9)

A first practical implication of the Act is the need to systematically identify and classify all AI systems in use. Once identified, each high-risk AI system must be integrated into a formal risk management process, with clear documentation and accountability structures. Hospitals should establish multidisciplinary governance committees to oversee AI deployment, ensuring that risk management, technical documentation, and human oversight are embedded within existing clinical governance and quality assurance frameworks (8, 21). Transparency obligations require hospitals to develop protocols for informing patients and staff about AI use. This is particularly important in high-stakes scenarios such as emergency triage or diagnostic support, where the rationale behind AI outputs must be clear and accessible. Training programs must be implemented to ensure that all relevant staff possess the necessary AI literacy to supervise, interpret, and, if necessary, override AI systems. The FRIA requires a new ethical and legal analysis, compelling hospitals to weigh the societal and individual consequences of AI deployment, particularly in areas of bias, discrimination, and data privacy. Furthermore, hospitals must prepare for ongoing regulatory engagement, including audits, reporting, and adaptation to evolving best practices and legal interpretations (22).

AI that is adaptive in nature introduces compliance complexity. Once deployed in a healthcare setting, vendor models may learn and evolve when exposed to real-world patient data, potentially altering their risk classification or performance profile. Under the EU AI Act, such changes may require updated risk management documentation, particularly where model drift could compromise clinical performance or patient safety.

As a result, in healthcare, the relationship between AI vendors and healthcare providers shifts from transactional procurement to ongoing compliance partnerships, ensuring sustained alignment on regulatory requirements, performance monitoring, and patient safety standards throughout the system's lifecycle.

### Step-by-Step compliance guidance

The Step-by-Step Compliance guide for healthcare facilities to comply with the EU AI Act is presented in the following. A practical example of an oncology center implementing a (hypothetical) multimodal AI-system for breast cancer is used for demonstration.

An additional section discussing In-House development of AI systems is provided in the Supplementary Appendix.

**Figure 2:**
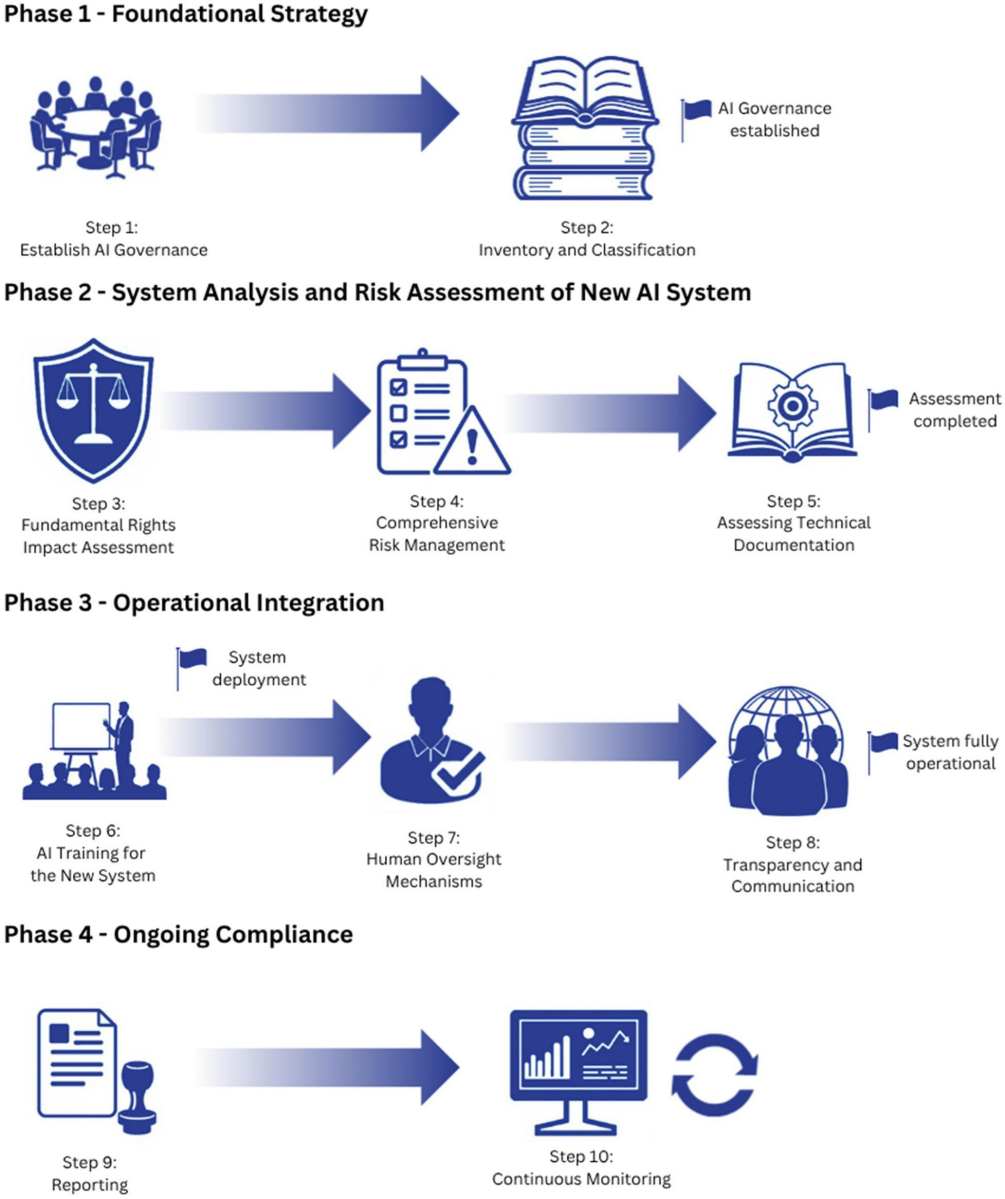
Schematic overview of the step-by-step compliance guide for the EU AI Act.

Figure 2 provides a schematic overview of this 10-step process, organized into four distinct phases: Foundational Strategy, Analysis & Risk Assessment, Operational Integration, and Ongoing Compliance. This visualization highlights the iterative nature of the framework, showing how foundational governance (Phase 1) informs the continuous monitoring and incident reporting required in the final phase (Phase 4). A practical handout summarizing the operational tasks and legal mandates for each step is provided in Supplementary File S1.

*Example: An oncology center decides to purchase a multimodal AI-system for clinical decision-support in patients with advanced breast cancer. The system called “BreastCancer-AI” uses multimodal data (imaging, pathology, and clinical) to classify patients into risk categories and suggest the best therapy sequence. It is being used to support case evaluation at the tumor board.*

#### Phase 1: foundational strategy

##### Step 1: establish AI governance

Establishing a robust foundation for AI compliance begins with strategic governance and a comprehensive understanding of all AI systems within a healthcare facility. This typically involves forming a multidisciplinary AI governance committee. Although the EU AI Act does not explicitly mandate the internal structure of a facility's governance, adopting a multidisciplinary approach (including clinical, legal, and IT leads) is a vital best-practice recommendation to meet the legal requirements of human oversight (Art. 26). This committee is responsible for setting and overseeing policies related to AI deployment, monitoring, and decommissioning. Overall aim is to ensure alignment with both the EU AI Act and existing MDRs as well as fostering a culture of transparency and accountability. A helpful guideline has been developed in the FUTURE-AI Framework for trustworthy and deployable AI in healthcare (8). The governance structures should be dynamic and responsive, with regular meetings, clear reporting lines to hospital leadership, and mechanisms for stakeholder engagement, including patient representatives where appropriate. This committee also establishes formal communication channels and service level agreements with AI vendors to ensure clear responsibilities and support.

*Example: The oncology center's AI Governance Committee is led by a Clinical Lead (Chief Oncologist) and a Technical Lead (Data Scientist). It also includes legal counsel and a patient advocate. Their concrete actions include:*
  - *Reporting: A quarterly dashboard is sent to the hospital board, tracking the “BreastCancer-AI” system's performance, clinician override rates, and any near-miss incidents.*
  - *Transparency: A patient-facing FAQ on the hospital website explains in simple terms what the AI does, clarifying that the final decision always rests with the human oncologist.*
  - *Oversight: A senior physician uses a structured log to manage staff-reported issues (e.g., “unexpected output,” “confusing explanation”) for monthly committee review.*
  - *Vendor Management: A service-level agreement (SLA) legally requires the AI vendor to provide a root cause analysis within 48 hours for any high-priority incident.*

##### Step 2: inventory and classification

Once governance structures are established, the next critical task is to conduct a thorough inventory and classification of all AI systems in use. This process extends beyond obvious clinical applications, such as diagnostic imaging or predictive analytics, to include AI within electronic health records (EHRs), administrative workflows, and patient engagement platforms. Each system must be evaluated against the definitions and risk categories set out in the EU AI Act. Particular attention should be given to identifying high-risk systems as defined in Article 6 and Annex III. Tools like the EU AI Act Compliance Checker (9) can support this process by providing tailored checklists and compliance pathways. Accurate classification is essential, as it determines the specific regulatory obligations that follow. Equally critical is a formal vendor due diligence process. Before a contract is signed, the healthcare facility as the deployer has a legal duty to verify that the high-risk AI system is accompanied by a valid EU declaration of conformity and the required technical documentation (Art. 26).

*Example: The committee evaluates the software systems currently in use and adds the “BreastCancer-AI” system to the existing inventory. Before finalizing the purchase, the legal and IT leads perform a formal due diligence audit. They verify the vendor's CE mark under MDR and ensure the vendor provides a “Compliance Package” including the instructions for use required for the hospital to meet its Art. 26 oversight obligations. They then use the “EU AI Act Compliance Checker” for final evaluation. The system is formally classified as “High Risk,” which requires full regulatory compliance.*

#### Phase 2: analysis & risk assessment of new AI system

##### Step 3: fundamental rights impact assessment

With established inventory and governance structure, healthcare facilities must conduct an FRIA for every high-risk AI deployment. The FRIA systematically evaluates the potential impacts of AI on privacy, non-discrimination, and equitable access to care.

This is a critical area of shared responsibility. While the AI provider is responsible for designing a fair and robust system as part of its CE marking process, the EU AI Act places a distinct obligation on the deployer (the hospital) to evaluate how that system will perform in its unique clinical context and with its specific patient population. A CE mark is a prerequisite, not a substitute, for the deployer's own due diligence.

The hospital's AI Governance Committee must therefore actively examine the system's limitations. This involves scrutinizing the provider's technical documentation and identifying potential risks that may only emerge upon deployment in their specific environment. The results must be thoroughly documented to ensure the AI's use aligns with both legal and ethical standards (4). It is important to note that the FRIA should be conducted proportionately: a highly autonomous triage AI requires an exhaustive analysis of potential discrimination, whereas a diagnostic support tool with a high degree of human oversight may allow for a more streamlined assessment focused primarily on data privacy and clinical accuracy.

Crucially, the FRIA must address the GDPR interface. Healthcare facilities must identify a lawful basis (typically Art. 6 and Art. 9 GDPR) for processing sensitive health data through the AI. This includes defining roles (is the hospital a sole controller or joint controller with the vendor?), setting clear data retention periods for AI inputs/outputs, and addressing secondary use (e.g., if the vendor wants to use hospital data to retrain their model). Issues of cross-border data transfers must be audited, particularly for cloud-based AI providers.

*Example: For “BreastCancer-AI,” the oncology center conducts a comprehensive FRIA that bridges clinical safety and data governance. The Data Protection Officer (DPO) verifies the lawful basis for processing (Art. 6 and 9 GDPR) and confirms a Data Processing Agreement (DPA) is in place, specifically auditing the vendor's cloud architecture to prevent unauthorized cross-border data transfers. The Technical lead confirms that patient data is pseudonymized before transmission and that a strict retention policy is set. Identifiable AI outputs are deleted after the statutory medical record period, while pseudonymized performance logs are kept for regulatory audit.*
*The technical documentation reveals the AI was trained primarily on North American and European populations. This flags a risk of lower performance for patients of Asian or African descent, who are common in the hospital's patient base. While the committee cannot change the model, they manage this risk operationally. The potential for reduced performance in specific demographic groups is formally documented and explicitly included in the mandatory training for all clinical users. Clinicians are instructed to apply extra scrutiny and rely on their professional judgment for these patient groups, with all overrides logged to monitor for patterns of failure or demographic bias.*

##### Step 4: comprehensive risk management

Comprehensive risk management is a fundamental concept of the EU AI Act. Articles 9–15 mandate a lifecycle-spanning risk management system for all high-risk AI systems. This process begins with a detailed risk assessment, identifying hazards from both intended use and foreseeable misuse, such as diagnostic errors, algorithmic bias, and cybersecurity threats. Risk management is inherently iterative, requiring continuous monitoring and updates as new data or incidents arise. Ideally, a multidisciplinary team should oversee this process, ensuring data representativeness, fairness, and regular audits for robustness and reliability (8). Providers of high-risk AI systems are required to register their systems in a central EU-wide database before market placement (Article 49), enhancing transparency and market surveillance. Deployers (healthcare facilities) also have obligations for post-market monitoring, including the reporting of any serious incidents or malfunctions to the provider [Article 26 (5)] and, where appropriate, to national market surveillance authorities (Article 72).

Cybersecurity is a critical component of risk assessment, with the Act requiring technical and organizational measures to prevent unauthorized access, data breaches, and adversarial attacks. This includes regular penetration testing, encryption of sensitive data, and incident response protocols. All risk management activities must be documented and integrated into the facility's broader quality and safety systems, ensuring traceability and accountability for all risk-related decisions (22).

*Example: For the “Breast Cancer AI” system, the oncology center's multidisciplinary risk management team initiates an evaluation process. They identify hazards like the AI misclassifying a patient or underperforming for specific ethnic groups. To mitigate cybersecurity threats, the IT security specialist implements a multi-layered strategy beyond basic encryption, including strict role-based access control (RBAC) and placing the AI on a segmented network to contain potential breaches. An incident response plan is established to ensure continuity of care if the system becomes unavailable. The “Breast Cancer AI” system has a continuous monitoring dashboard to track the concordance rate between the AI's therapy suggestions and the final decisions of the tumor board. This performance data is stratified by patient demographics to audit for bias, with bi-annual reviews to detect any model drift or new risks.*

##### Step 5: assessing technical documentation

A critical task for the deployer is to assess the technical documentation provided by the AI system's developer. Under Articles 11 and 18, the provider is legally required to supply a comprehensive technical file. The healthcare facility, as the deployer, has to review this document for completeness and clarity, and maintain it as part of its own compliance records. The hospital's AI Governance Committee, specifically its members with data science or technical expertise, must understand the system's intended purpose, performance metrics, known limitations, and risk mitigation strategies. This review is not a one-time event and must repeated for substantial software updates (23).

Traceability is crucial. The provider is responsible for logging all updates, retraining, and modifications. When the hospital receives a software update from the vendor (for instance, one incorporating new clinical guidelines) it must ensure it also receives the updated technical documentation and validation reports. Deploying an updated version without this documentation would be a serious compliance failure. Furthermore, technical documentation is only the starting point. Healthcare facilities must conduct “Local Pre-Go-Live Validation”. This involves: Site-specific testing to ensure the AI performs consistently on local hardware, checks to identify if the AI performs as expected in the local environment, and interoperability testing to ensure seamless data flow between the AI and existing EHR or PACS systems.

*Example: For the “BreastCancer-AI” system, the oncology center received the complete technical file from the vendor. Before clinical launch, the medical physics and IT teams conduct a “Local Validation Study”. They run 100 historical local cases through the AI to verify that its accuracy matches the vendor's claims when used with the hospital's specific pathology slides and MRI protocols. They specifically perform “subgroup checks” to confirm performance for elderly patients (>80 years), a demographic often underrepresented in training data. Finally, an “interoperability audit” ensures that AI suggestions are automatically pulled into the Tumor Board's digital dashboard, preventing manual transcription errors. The AI Governance Committee's technical lead (the data scientist) reviews the technical document, paying special attention to the validation studies. They verify that the performance metrics are clearly defined and that the documentation transparently discloses known limitations, such as reduced performance for rare breast cancer histologies.*
*After the system had already been operational for several months, the vendor releases a new version which has been updated to reflect new ASCO guidelines. The hospital's protocol dictates that the update is not deployed immediately. Instead, the committee first obtains the revised technical file. The technical lead reviews the new validation report to confirm the update has not negatively impacted performance before the new version is approved for deployment.*

#### Phase 3: operational integration

##### Step 6: training and AI literacy

With comprehensive system analysis, risk assessments, and documentation reviews complete, the focus shifts to the user. Before the AI is deployed in a live clinical setting, it is essential to ensure that healthcare professionals have system-specific adequate AI literacy. Training programs must translate the findings from the previous steps into practical knowledge for clinicians. They should cover the system's specific capabilities and, crucially, the limitations and risks identified during the FRIA and risk management phases. This includes training on the legal and ethical obligations under the EU AI Act. A hybrid approach is effective, combining system-specific knowledge from the provider with institution-specific training on local clinical workflows and override protocols. Scenario-based training and regular refresher courses are vital to help staff develop the skills needed to supervise, interpret, and, when necessary, confidently override AI outputs.

While no formal certification is mandated by the Act, healthcare facilities must be able to demonstrate staff competence through robust training records and assessments (24).

*Example: Following the AI Governance Committee's formal approval of the “BreastCancer-AI” system based on the completed risk assessments, the hospital launches a mandatory, pre-deployment training program for all oncology staff. The training uses the vendor's materials but is customized with hospital-specific content. For instance, it emphasizes the risk of lower performance for certain ethnic groups (as identified in the FRIA) and instructs clinicians on the protocol for applying extra scrutiny in those cases. The training uses hands-on sessions with test cases provided by the vendor that showcase both successes and known failure modes, ensuring staff can interpret confidence intervals and know when to question the AI's recommendations.*

##### Step 7: human oversight mechanisms

Human oversight, as required by Articles 14 and 26 (2), is a legal obligation. We recommend as best practice the use of “explainability dashboards” and structured override protocols to mitigate cognitive biases like automation bias. Meaningful oversight requires critical evaluation of the AI-generated recommendation within the full clinical context. Healthcare facilities must designate qualified personnel to supervise each high-risk system. Oversight involves staff recognizing unreliable or inappropriate outputs, such as when a diagnostic tool produces an unexpected result. Oversight personnel must have the authority and technical means to adapt or deactivate the AI system, if necessary. This is particularly important in situations where patient safety is at risk. A primary goal of human oversight must be to mitigate predictable cognitive biases:

- **Confirmation Bias:** The tendency for clinicians to favor the AI's conclusion when it aligns with their own initial assessment and dismiss it when it does not.
- **Automation Bias:** The tendency to over-rely on the AI's suggestion and accept it without sufficient scrutiny, even when it contradicts other evidence or a clinician's own judgment.

Effective oversight mechanisms and training must empower clinicians to achieve calibrated trust, neither reflexively dismissing the AI nor blindly accepting its outputs.

The Act requires that AI systems provide understandable explanations for their outputs, enabling clinicians to make informed decisions and communicate effectively with other clinicians as well as with patients.

Human oversight should also be a crucial part of the facility's broader clinical governance framework. By institutionalizing human oversight, healthcare facilities can ensure that AI systems augment, rather than replace or hinder, clinical judgment, thereby safeguarding patient safety and upholding ethical standards (25).

*Example: For the “BreastCancer-AI” system the hospital's oversight protocol is integrated directly into the multidisciplinary tumor board's workflow. The AI's “explainability dashboard” is designed to be clinically actionable; it presents a confidence score for its recommendation and highlights cases where a patient's data profile is an outlier compared to the training data. To mitigate confirmation bias, the tumor board's standard procedure requires that if the AI's recommendation differs from the initial consensus, its rationale must be explicitly discussed. Simultaneously, to counter automation bias, clinicians are trained to ensure that overriding the AI is a normal and expected part of the process, with all final decisions remaining the sole responsibility of the human clinician. Emergency protocols are in place, allowing the designated AI Clinical Lead to immediately disable the system's recommendation feature, reverting it to a data-display-only mode, if any systematic errors or safety concerns are detected.*

##### Step 8: transparency and communication

Transparency is a foundational requirement of the EU AI Act. Healthcare facilities must develop protocols for informing staff as well as patients about the use of AI, including the purpose, implementation and limitations of AI-driven decisions. This involves updating consent forms, patient information leaflets, and staff training materials to reflect the presence and role of AI in clinical workflows. The Act also requires that synthetic content generated by AI, such as automated reports or patient communications, be marked as such. In addition, facilities must be prepared to provide explanations of AI outputs upon request, supporting both patient autonomy and clinical accountability.

*Example: Patients receive updated consent forms mentioning the role of the “BreastCancer-AI” system. The hospital creates infographics showing how AI combines data types for predictions. Patient reports include disclaimers about probabilistic uncertainty. Clinicians explain that “BreastCancer-AI” augments but doesn't replace medical judgment. The hospital website features a dedicated AI page with information and patient rights information on all the AI systems used in the clinic. The oncology center collaborates with the vendors of the AI systems to ensure patient-facing materials accurately reflect the AI's capabilities and limitations as per the provider's specifications and regulatory approvals.*

#### Phase 4: ongoing compliance

##### Step 9: registration and reporting

Compliance with the EU AI Act is an ongoing commitment that extends throughout the lifecycle of each AI system. While the provider is legally responsible for registering a high-risk AI system in the EU database, the deployer's (the facility's) obligation is to verify this registration before use [Art. 26 (8)]. Furthermore, comprehensive logs must be maintained to support audit and investigation processes. Facilities should establish protocols for regular reporting, including incident reporting and periodic compliance reviews. This ensures ongoing regulatory engagement and facilitates rapid response to emerging risks or regulatory changes (26).

*Example: The “BreastCancer-AI” system is registered with national health authorities with regular compliance reports. An incident reporting system integrates with patient safety frameworks. The first quarterly report identifies three near-misses caught by human review. Documentation complies with MDRs and GDPR requirements. The oncology center establishes a direct reporting channel with the vendor for critical incidents and performance anomalies, ensuring the vendor is promptly informed of issues affecting the AI system in real-world clinical use, aiding in their own post-market surveillance.*

##### Step 10: continuous monitoring

Continuous monitoring and quality assurance are needed for safe AI integration. “Continuous” implies an ongoing, systematic process of observing, analyzing, and adapting the AI system's performance and compliance throughout its lifecycle, employing both automated data collection and regular, risk-based reviews. The frequency of checks should be determined by the AI system's risk level, clinical impact, and dynamism. Healthcare facilities must establish processes for ongoing performance monitoring, regular updates to technical documentation, and adaptation to evolving best practices. This includes staying informed about regulatory updates, participating in professional networks, and engaging with external audits or peer reviews. By integrating continuous monitoring, hospitals can ensure that AI systems remain safe, effective, and aligned with regulatory and clinical standards (26).

*Example: Regular quality assurance reviews compare predictions against 6-month patient outcomes. Performance metrics are stratified by cancer type, stage, and demographics to detect biases. The team participates in a “European oncology AI network” sharing anonymized performance data. Vendor updates undergo validation on internal historical cases before deployment. One year after the deployment, monitoring shows improved performance after vendor-provided updates, but it also identifies gaps in post-immunotherapy patients. These insights are shared with the vendor for potential future system enhancements.*

## Discussion

### Interpretation of findings in relation to research objectives

Analyzing the EU AI Act emphasizing the perspective of healthcare facilities as AI deployers, the several aspects are relevant for the aforementioned three research questions of this work.

- Question 1 (Legal and Ethical Obligations): Our analysis reveals substantial responsibilities transitioning from the developer to the healthcare facility once the system is deployed. While the Act mandates technical safety from providers, large parts of the ethical burden, specifically regarding patient-centered transparency and fundamental rights (Art. 27) rests primarily with the clinical deployer.
- Question 2 (Governance Adaptation): The 10-step roadmap demonstrates that existing hospital governance is currently in many situations too siloed to meet the Act's requirements. Our synthesis of Phase 1 (Foundational Strategy) interprets the Act's “human oversight” mandate as a requirement for a new, integrated multidisciplinary committee model that does not currently exist in most healthcare facilities (see also Figure 2).
- Question 3 (Operational Lifecycle): Through the “BreastCancer-AI” example, we interpret the Act's requirement for “continuous monitoring” as a clinical quality assurance mandate. Compliance appears not to be a “one-time CE-mark check” but a dynamic process requiring active clinician engagement to mitigate automation bias throughout the system's entire lifecycle.

### Comparative analysis and scientific contribution

While the legal architecture of the EU AI Act or its technical implications for AI developers has extensively been discussed previously, little work focused specifically on the operational duties of healthcare facilities. For instance, while van Kolfschooten and van Oirschot provided a comprehensive overview of the Act's health-related legal risks (22), their work primarily addresses the regulatory theory without providing an implementation guidance. Our work contributes to the field by providing a framework for implementation of AI according to the legal requirements in clinical workflows. It transitions from regulatory analysis to an actionable governance model, providing an orientation that balances legal compliance with the practical realities of a high-pressure clinical environment.

### Limitations, shortcomings and challenges of the EU AI Act

Despite its intent to foster trustworthy AI, the practical application of the EU AI Act reveals several possible shortcomings and inherent challenges, particularly for healthcare innovators and deployers. Indeed, the aforementioned recent EU Study on AI deployment in healthcare noted that 72% of healthcare professionals believe the Act creates new challenges related to implementation (5). A primary concern mentioned by stakeholders is that the comprehensive compliance burden imposed by the Act, especially on deployers, risks undermining a core purpose of AI in healthcare: to enhance efficiency and ease workload. Concerns are that the Act could create more administrative overhead than clinical benefit.

Furthermore, there is still a lot of uncertainty and confusion, a problem that is also linked to the basic definitions. While the EU AI Act provides a definition of an “AI system” (Art. 3), its breadth means it does not always clearly enough delineate what an AI-based system is in practice, or which specific technologies are considered AI. This may create confusion among providers, regulatory bodies, and implementers. For instance, systems that employ Bayesian statistical principles or other non-deterministic mathematical methods are often considered forms of AI. Also, fully deterministic systems based on knowledge-based technologies can be regarded as forms of AI. What constitutes an “AI system” is not definitively clear, making it difficult to determine which systems truly qualify.

From the providers perspective, the AI Act creates substantial hurdles, particularly for smaller innovative companies and startups, hindering their ability to develop potentially lifesaving solutions. This may substantially impact the European economic space, especially considering the latest deregulations in the United States, and the pace of development and implementation of AI-based systems in China and other countries.

Furthermore, the Act's static, approval-based framework struggles to address rapidly evolving technologies, particularly general-purpose AI and foundation models. For instance, retraining a model with new local data to improve its performance for a specific patient population could be classified as a “substantial modification” [Art. 3 (23)] under the Act. This would trigger a full re-certification process, a tremendous and often prohibitively expensive effort (Art. 25). Without more agile mechanisms for regulatory adaptation, key protections risk becoming outdated while beneficial updates are discouraged.

Finally, the Act's heavy reliance on concepts like “human oversight” and “AI literacy” raises serious questions about its real-world feasibility. The regulation does not provide a clear definition of the competencies required for “AI literacy.” Without pragmatic solutions, the obligation for meaningful oversight may become an unrealistic and unfunded mandate for already overburdened healthcare systems.

Despite considerable challenges and some limitations, the EU AI Act provides an essential framework for responsible and trustworthy integration of AI. While it is a European regulation, it remains to be seen whether it could become a de facto global standard similar to the GDPR. The comprehensive, risk-based approach of the EU AI Act is already shaping policy discussions in other jurisdictions, including the United States, Canada, and Asia-Pacific, where lawmakers are considering analogous frameworks for AI governance. For global healthcare technology vendors and multinational hospital systems, alignment with the EU AI Act is likely to become a prerequisite for market access and reputational credibility. Some jurisdictions may look to the Act as a reference point for high-standard AI governance, while others might pursue more sector-specific or voluntary frameworks. Nonetheless, for healthcare organizations operating internationally, aligning with these principles may offer a strategic advantage in ensuring patient safety and building cross-border trust in AI-driven care.

## Conclusion

By establishing clear, enforceable standards for risk management, transparency, technical documentation, and human oversight, the EU AI Act provides a framework for the safe and ethical deployment of AI in hospitals and clinics. The Act's emphasis on transparency, human oversight, and fundamental rights resonates with broader societal expectations and ethical imperatives, making it a model for responsible AI deployment.

For hospital administrators and clinicians, compliance with the Act is both a legal obligation and an opportunity to enhance patient safety, foster innovation, and build public trust. As AI continues to transform healthcare, the basic principles of the EU AI Act of risk-based governance, transparency, and respect for fundamental rights, will become increasingly important. Proactive engagement with these requirements will not only ensure regulatory compliance but also position healthcare organizations at the forefront of responsible, patient-centered innovation.

## References

[1] Hastings J. Preventing harm from non-conscious bias in medical generative AI. Lancet Digit Health. (2024) 6(1):e2–3. 10.1016/S2589-7500(23)00246-7 38123253

[2] Habli I Lawton T Porter Z. Artificial intelligence in health care: accountability and safety. Bull World Health Organ. (2020) 98(4):251–6. 10.2471/BLT.19.237487 32284648 PMC7133468

[3] Shoghli A Darvish M Sadeghian Y. Balancing innovation and privacy: ethical challenges in AI-driven healthcare. J Rev Med Sci. (2024) 4(1):1–11. 10.22034/jrms.2024.494112.1034

[4] EU Artificial Intelligence Act. Available online at: https://artificialintelligenceact.eu/the-act/ (Accessed April 2, 2026)

[5] European Commission. Directorate general for health and food safety., PwC., EEIG., open evidence. In: Study on the Deployment of AI in Healthcare: Final Report. LU: Publications Office (2025). Available online at: https://data.europa.eu/doi/10.2875/2169577 (Accessed November 3, 2025).

[6] EU. Medical Device Regulation (MDR 2017/745). Available online at: https://eur-lex.europa.eu/eli/reg/2017/745/oj/eng

[7] EU. General Data Protection Regulation (GDPR 2016/679). Available online at: https://eur-lex.europa.eu/eli/reg/2016/679/oj/eng

[8] Lekadir K Frangi AF Porras AR Glocker B Cintas C Langlotz CP. FUTURE-AI: international consensus guideline for trustworthy and deployable artificial intelligence in healthcare. Br Med J. (2025) 388:e081554. 10.1136/bmj-2024-081554 39909534 PMC11795397

[9] EU. Artificial Intelligence Act, Compliance Checker. Available online at: https://artificialintelligenceact.eu/assessment/eu-ai-act-compliance-checker/ (Accessed April 2, 2026)

[10] Kelly CJ Karthikesalingam A Suleyman M Corrado G King D. Key challenges for delivering clinical impact with artificial intelligence. BMC Med. (2019) 17(1):195. 10.1186/s12916-019-1426-2 31665002 PMC6821018

[11] Amann J Blasimme A Vayena E Frey D Madai VI. Precise4Q consortium. Explainability for artificial intelligence in healthcare: a multidisciplinary perspective. BMC Med Inform Decis Mak. (2020) 20(1):310. 10.1186/s12911-020-01332-6 33256715 PMC7706019

[12] Mennella C Maniscalco U De Pietro G Esposito M. Ethical and regulatory challenges of AI technologies in healthcare: a narrative review. Heliyon. (2024) 10(4):e26297. 10.1016/j.heliyon.2024.e26297 38384518 PMC10879008

[13] Al Kuwaiti A Nazer K Al-Reedy A Al-Shehri S Al-Muhanna A Subbarayalu AV. A review of the role of artificial intelligence in healthcare. J Pers Med. (2023) 13(6):951. 10.3390/jpm13060951 37373940 PMC10301994

[14] Dennstädt F Hastings J Putora PM Schmerder M Cihoric N. Implementing large language models in healthcare while balancing control, collaboration, costs and security. Npj Digit Med. (2025) 8(1):143. 10.1038/s41746-025-01476-7 40050366 PMC11885444

[15] Feng QJ Harte M Carey B Alqarni A Monteiro L Diniz-Freitas M. The risks of artificial intelligence: a narrative review and ethical reflection from an oral medicine group. Oral Dis. (2025) 31(2):348–53. 10.1111/odi.15100 39176474 PMC11976142

[16] Busch F Kather JN Johner C Moser M Truhn D Adams LC. Navigating the European union artificial intelligence act for healthcare. Npj Digit Med. (2024) 7(1):210. 10.1038/s41746-024-01213-6 39134637 PMC11319791

[17] European Parliament. Directorate general for parliamentary research services. In: Artificial Intelligence in Healthcare: Applications, Risks, and Ethical and Societal Impacts. LU: Publications Office (2022). Available online at: Available from: https://data.europa.eu/doi/10.2861/568473 (Accessed April 2, 2026)

[18] Almyranti M Sutherland E Ash N Eiszele S. Artificial Intelligence and the Health Workforce: Perspectives from Medical Associations on AI in Health [OECD Artificial Intelligence Papers]. 28th ed. Paris: OECD Artificial Intelligence Papers (2024). Available online at: https://www.oecd.org/en/publications/artificial-intelligence-and-the-health-workforce_9a31d8af-en.html (Accessed April 2, 2026)

[19] Bayani A Epoh Ewane LP Oliveira Dos Anjos DS Mac-Seing M Nikiema JN. Leveraging open-source large language models (LLMs) in scoping reviews: a case study on disability and AI applications. Int J Med Inf. (2025) 204:106048. 10.1016/j.ijmedinf.2025.106048 40729777

[20] EU. EUR-Lex: Regulation (EU) 2017/745 of the European Parliament and of the Council of 5 April 2017 on Medical Devices, Amending Directive 2001/83/EC, Regulation (EC) No 178/2002 and Regulation (EC) No 1223/2009 and Repealing Council Directives 90/385/EEC and 93/42/EEC (Text with EEA relevance.). Available online at: https://eur-lex.europa.eu/eli/reg/2017/745/oj/eng (Accessed April 2, 2026)

[21] Bouderhem R. Shaping the future of AI in healthcare through ethics and governance. Humanit Soc Sci Commun. (2024) 11(1):416. 10.1057/s41599-024-02894-w

[22] Van Kolfschooten H Van Oirschot J. The EU artificial intelligence act (2024): implications for healthcare. Health Policy. (2024) 149:105152. 10.1016/j.healthpol.2024.105152 39244818

[23] Mienye ID Obaido G Jere N Mienye E Aruleba K Emmanuel ID. A survey of explainable artificial intelligence in healthcare: concepts, applications, and challenges. Inform Med Unlocked. (2024) 51:101587. 10.1016/j.imu.2024.101587

[24] Schubert T Oosterlinck T Stevens RD Maxwell PH Van Der Schaar M. AI education for clinicians. eClinicalMed. (2025) 79:102968. 10.1016/j.eclinm.2024.102968 PMC11667627 39720600

[25] Rosenbacke R Melhus Å McKee M Stuckler D. How explainable artificial intelligence can increase or decrease Clinicians’ trust in AI applications in health care: systematic review. Jmir AI. (2024) 3:e53207. 10.2196/53207 39476365 PMC11561425

[26] Babic B Glenn Cohen I Stern AD Li Y Ouellet M. A general framework for governing marketed AI/ML medical devices. Npj Digit Med. (2025) 8(1):328. 10.1038/s41746-025-01717-9 40450160 PMC12126487

