# Supplementary material for: The EU AI Act: implications and compliance guidance for healthcare facilities
Source: Front Digit Health. 2026 Jun 10;8:1808373. doi: 10.3389/fdgth.2026.1808373 (PMC13292296; doi:10.3389/fdgth.2026.1808373)
Supplement: Supplementary file 1 [file Table1.docx]

| **Domain** | **Obligations of Providers** | **Obligations of Deployers** |
| --- | --- | --- |
| **Risk Management System** (Art. 9) | Establish, implement, maintain, and update a comprehensive risk management system for the AI system throughout its entire lifecycle. | Use the AI system according to the provider's instructions for risk management (Art. 26(1)). Inform the provider of any identified risks (Art. 26(5) and Art. 72). |
| **Data Data Governance** (Art. 10) | Ensure high quality of training, validation, and testing datasets (relevant, representative, free of errors, complete). Implement measures to address biases. | Ensure input data used during operation is relevant and representative (Art. 26(4)). |
| **Technical Documentation** (Art. 11) | Draw up and keep comprehensive technical documentation demonstrating compliance. Keep it updated for 10 years after the AI system is placed on the market. | Obtain documentation from the provider, review it for completeness and clarity, and maintain it as part of its own compliance records. |
| **Record-keeping (Logging)** (Art. 12) | Design AI systems to automatically generate logs during operation to facilitate monitoring and traceability. | Ensure the logging functionality is working and keep the logs for a period appropriate to the system's purpose (Art. 26(6)). |
| **Transparency & Instructions for Use** (Art. 13) | Design AI systems for appropriate transparency and provide clear, comprehensive instructions for use. | Use the AI system in accordance with the instructions for use (Art. 26(1)). Inform affected persons (e.g., patients/staff) that they are interacting with an AI system. |
| **Human Oversight** (Art. 14) | Design AI systems to be effectively overseen by natural persons, including enabling human intervention and override. | Ensure that the AI system is subject to effective human oversight by the staff. Establish oversight processes and provide necessary training (Art. 26(2)). |
| **Accuracy, Robustness & Cybersecurity** (Art. 15) | Design and develop AI systems to achieve appropriate levels of accuracy, robustness, and cybersecurity. | Operate the AI system with the appropriate technical and security measures. Monitor for performance drops or cybersecurity issues (Art. 26(1)). |
| **Quality Management System** (Art. 17) | Implement a quality management system to ensure compliance with the AI Act. | Integrate AI deployment into the facility's existing quality management and clinical governance frameworks. |
| **Fundamental Rights Impact Assessment (FRIA)** (Art. 27) | Provide necessary information and support to deployers to enable them to conduct their FRIAs. | Before putting the system into use, conduct an assessment of the impact on fundamental rights. |
| **Conformity Assessment & CE Marking** (Art. 43-46) | Perform a conformity assessment and affix the CE marking to the AI system. | Verify that the high-risk AI system bears the CE marking and is accompanied by the EU declaration of conformity. |
| **Registration in EU Database** (Art. 49) | Register the high-risk AI system in the EU database. | Deployers who are public authorities or acting on their behalf must register their use of the high-risk AI system in the database (Arts. 26(8), 49). |
| **Post-Market Monitoring** (Art. 61) | Establish a system to continuously collect and analyze data on the AI system's performance and any serious incidents. | Cooperate with the provider and provide relevant data from their use concerning any serious incidents or performance changes (Arts. 26(5), 72). |
| **Serious Incident Reporting** (Art. 62) | Report any serious incidents or malfunctions to the relevant national market surveillance authorities. | Report any serious incidents they become aware of to the provider and/or relevant national authorities (Arts. 26(5), 72). |
| **Corrective Actions** (Art. 65) | Take immediate corrective actions if the AI system is found to be non-compliant or poses a risk. | Cooperate with the provider and authorities on necessary corrective actions. Suspend use if significant risks arise. |
| **Cooperation with Authorities** (Art. 68) | Cooperate with market surveillance authorities, providing information and facilitating inspections. | Cooperate with market surveillance authorities, providing information and facilitating inspections. |
| **AI Literacy & Training** | Provide adequate training and information on the specific AI system to enable safe and compliant use by deployers. | Ensure all relevant staff receive adequate AI literacy training. Maintain training records (Art. 26(2)). |
| **Supply Chain & Partnerships** | Engage in ongoing compliance partnerships with deployers, providing updates and support throughout the AI system's lifecycle. | Establish ongoing compliance partnerships with providers to ensure alignment on regulatory and safety standards. |

Supplementary Table 1: Summary of obligations of providers and deployers of high-risk AI systems applicable to healthcare deployment
