# Supplementary material for: The EU AI Act: implications and compliance guidance for healthcare facilities
Source: Front Digit Health. 2026 Jun 10;8:1808373. doi: 10.3389/fdgth.2026.1808373 (PMC13292296; doi:10.3389/fdgth.2026.1808373)
Supplement: Supplementary file 2 [file Supplementaryfile1.docx]

**Supplementary File – Handout for the EU AI Act Compliance Guide for Healthcare Facilities**

| **Step** | | **Description** | **Key Tasks** |
| --- | --- | --- | --- |
| ***Phase 1: Foundational Strategy*** | | | |
| 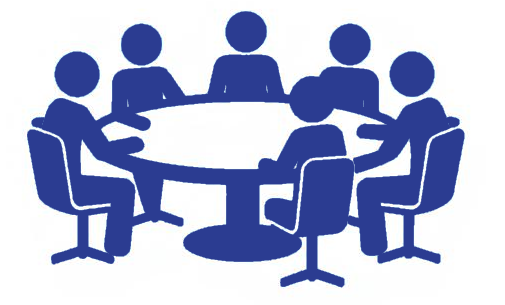 | 1. Establish AI Governance | Creating the institutional framework for oversight and accountability. | • Form a multidisciplinary AI Committee (Clinical, IT, Legal, Ethics).  • Define reporting lines to hospital leadership.  • Establish Service Level Agreements (SLAs) with AI vendors. |
| 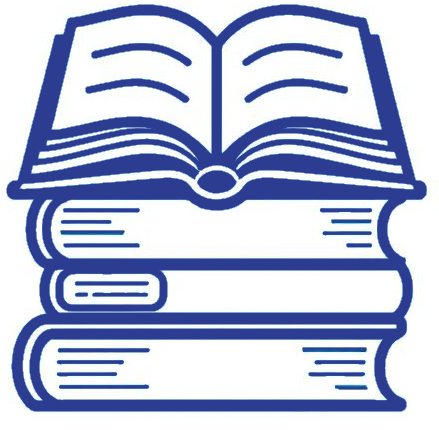 | 2. Inventory & Classification | Identifying all AI systems and determining their regulatory risk level. | • Map all AI-enabled software in clinical and administrative use.  • Classify systems (High-Risk, Limited, etc.) per Art. 6 and Annex III.  • Conduct vendor due diligence for CE marking and EU registration. |
| ***Phase 2: Analysis & Risk Assessment*** | | | |
| 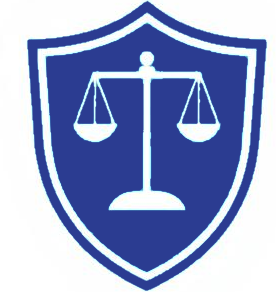 | 3. Fundamental Rights Impact Assessment (FRIA) | Evaluating the socio-ethical impact of AI system on the specific patient population. | • Analyze risks to privacy, non-discrimination, and equitable access.  • Audit the GDPR lawful basis for processing sensitive health data.  • Document site-specific mitigation strategies for identified biases. |
| 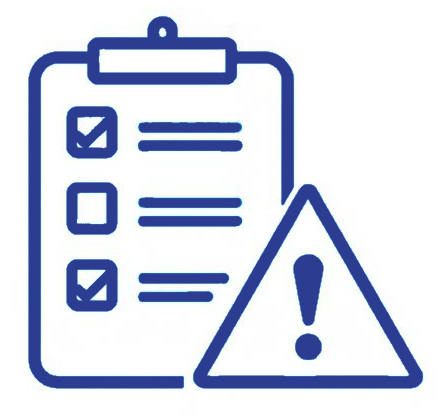 | 4. Comprehensive Risk Management | Integrating AI risks into the existing hospital safety and quality systems. | • Identify hazards from intended use and foreseeable misuse.  • Implement multi-layered cybersecurity (encryption, access control).  • Create a clinical continuity plan for AI system downtime. |
| 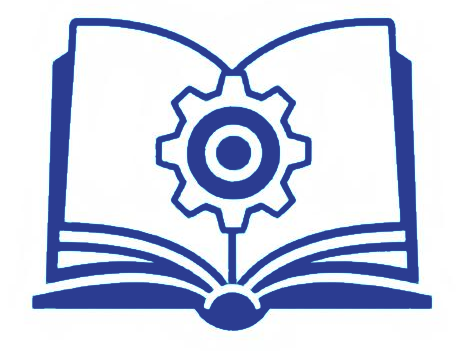 | 5. Technical Documentation & Validation | Verifying the provider's technical claims within the local clinical environment. | • Review the vendor's technical file for clarity and completeness.  • Perform "Local Pre-Go-Live Validation" on local hardware and data.  • Ensure interoperability with existing EHR and PACS systems. |
| ***Phase 3: Operational Integration*** | | | |
| 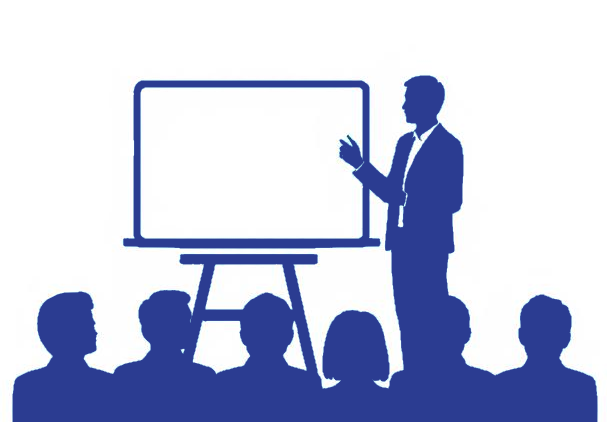 | 6. Training and AI Literacy | Empowering staff to use AI safely and recognize its limitations. | • Provide mandatory system-specific training for clinical users.  • Educate staff on "black box" risks and legal/ethical obligations.  • Maintain robust training records to demonstrate staff competence. |
| 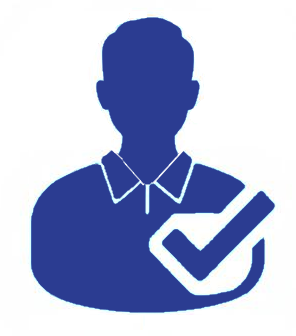 | 7. Human Oversight Mechanisms | Ensuring human judgment remains the final safeguard in the clinical loop. | • Implement "Explainability Dashboards" to support clinician trust.  • Establish formal override protocols to mitigate automation bias.  • Assign qualified personnel to supervise real-time AI operations. |
| 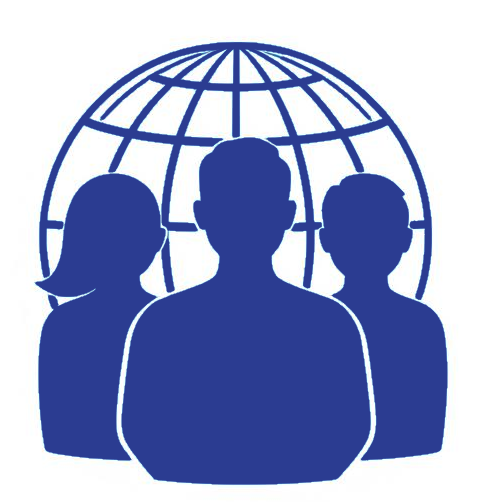 | 8. Transparency and Communication | Informing stakeholders about the role of AI in medical decision-making. | • Update patient consent forms and information leaflets.  • Clearly label AI-generated content (e.g., automated reports).  • Develop protocols to provide "meaningful explanations" upon request. |
| ***Phase 4: Ongoing Compliance*** | | | |
| 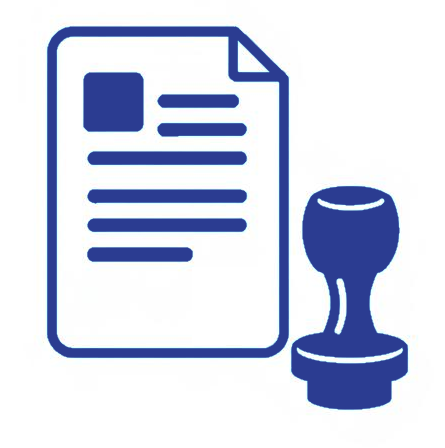 | 9. Registration and Reporting | Maintaining regulatory engagement throughout the system's lifecycle. | • Verify the AI system's presence in the central EU database.  • Maintain operational logs for audit and investigation (min. 6 months).  • Report serious incidents or malfunctions to vendors and authorities. |
| 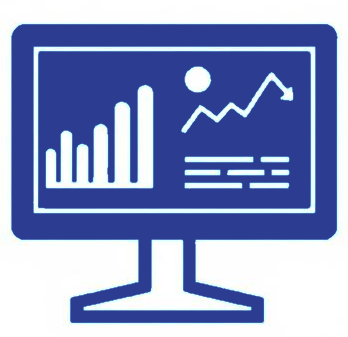 | 10. Continuous Monitoring | Iteratively assessing performance to prevent model drift or safety gaps. | • Schedule regular quality assurance audits against clinical outcomes.  • Monitor concordance between AI suggestions and clinician decisions.  • Re-validate the system following "substantial updates" or retraining. |
