# Supplementary material for: The EU AI Act: implications and compliance guidance for healthcare facilities
Source: Front Digit Health. 2026 Jun 10;8:1808373. doi: 10.3389/fdgth.2026.1808373 (PMC13292296; doi:10.3389/fdgth.2026.1808373)
Supplement: Supplementary file 3 [file Supplementaryfile2.docx]

Special Considerations: In-House Development of AI Systems

While this guide focuses on deploying an AI system developed by an external company, for some situations healthcare facilities may also consider own system development. Healthcare institutions may develop and use in-house developed AI tools for clinical applications under Article 2, paragraph 8, provided they meet all regulatory requirements, including compliance with general safety and performance requirements (Annex I) and maintain an appropriate quality management system. Healthcare facilities retain autonomy to develop in-house solutions when they can justify that specific patient needs, performance requirements, integration necessities, or institutional workflows cannot be adequately addressed by commercially available products.

However, before developing an in-house device, institutions must examine the market for equivalent CE-marked devices and document justification that the target group's specific needs cannot be met at the appropriate level of performance by available alternatives. This justification must consider technical and clinical equivalence.

Institutions must continuously monitor the market for potentially equivalent devices. Suppose an equivalent CE-marked device becomes available that meets the patient group's needs at the appropriate level of performance. In that case, the health institution should review its justification and may initiate a transition process toward the CE-marked device.
